# Effects of Two Composite Modifications on the Emulsifying and Potential Gel Properties of Palm Kernel Cake Glutelin-1

**DOI:** 10.3390/gels12030229

**Published:** 2026-03-11

**Authors:** Peiyao Long, Ling Dang, Zhihui Wei, Zihao Zhang, Yajun Zheng, Hao Wang, Jiaying Pei

**Affiliations:** 1 Food Science College, Shanxi Normal University, Taiyuan 030092, China; lpy166357173632023@163.com (P.L.); 17836564558@163.com (Z.W.); 17635463062@163.com (Z.Z.); 17305306932@163.com (H.W.); 18406536770@163.com (J.P.); 2 School of Health Management, Shanxi Technology and Business University, Taiyuan 030006, China

**Keywords:** oil palm kernel cake glutelin-1, ultrasonication, gallic acid conjugation, arabinose glycosylation, emulsifying properties, mechanism insights

## Abstract

Palm kernel cake glutelin-1 (PKCG-1) can be used as a novel emulsifier, contingent upon enhancement of its emulsifying functionality. This study investigated the influences and underlying mechanisms of ultrasonication-assisted gallic acid-binding or arabinose-glycosylation on the emulsifying properties of PKCG-1. The results demonstrated that ultrasonication-assisted gallic acid-binding yielded the greatest improvement in emulsifying ability (from 91.03 to 159.74 m^2^/g), attributed to a concomitant decrease in molecular mass (from 59.2 to 48.4 kDa); increases in hydrophobicity (from 681 to 770), random coil content, and interfacial adsorption capacity (from 102.62 to 244.41 μg/mL); a reduction in the emulsion’s loss factor; and augmentation of zeta-potential (from −39.55 to −65.96 mV) and centrifugal stability (from 57.80% to 84.14%). Alternatively, ultrasonication-assisted arabinose glycosylation was best at enhancing the emulsion stability of PKCG-1 (from 79.77% to 98.36%) by increasing its solubility (from 28.35 to 73.85 g/100 mL) and random coil (from 25.9% to 46.9%), enhancing zeta-potential (from −39.55 to −84.81 mV) and viscosity; and reducing droplet size (1.10 to 0.64 μm) and loss tangent. Furthermore, the solubility, emulsifying activity, and emulsion stability of PKCG-1 decreased as pH increased from 2.5 to 8.5. Nevertheless, the application of the modified PKCG-1s as gels requires further studies.

## 1. Introduction

Emulsifiers are widely used in food systems containing both continuous and dispersed phases, as they can facilitate the formation of stable emulsions in which hydrophilic and hydrophobic substances stably coexist [1]. Emulsifiers play a crucial role in the processing, transportation, digestion, and absorption of nutrients such as lipids, fat-soluble vitamins, and cholesterol [2]. In recent decades, food protein-based emulsifiers have received increasing attentions for their advantages in safety, nutritional value, antioxidant activity, environmental sustainability, availability, and cost-effectiveness compared to chemically synthesized emulsifiers [3]. The preparation, characterization, and applications of food protein-based emulsifiers in fat-soluble bioactive substances (such as curcumin, carotene, and vitamin E) have been widely investigated [4,5,6]. However, plant protein-based emulsifiers have some drawbacks, such as relatively lower emulsifiability and emulsion stability, poor solubility or hydrophobicity, and a susceptibility to environmental factors [7]. To address these limitations, various chemical, biological, and physical approaches have been employed to enhance the emulsifying properties of plant proteins [8,9,10]. Glycosylation can enhance the emulsifying properties of proteins by increasing their solubility and interface adsorption capacity [11]. Arabinose is commonly found in the form of polysaccharides in plant fruit pulps, colloids, hemicellulose, pectic acids, bacterial polysaccharides, and certain glycosides [12]. Prior studies have demonstrated that protein–arabinose conjugate have considerable solubility, emulsifying and foaming properties [13,14]. Moreover, the grafting of polyphenols has been shown to improve the hydrophobicity and interfacial adsorption capacity of proteins, thereby enhancing their emulsifying and antioxidant performance [2,8]. Ultrasonication displays various effects such as mechanical vibration, cavitation, and thermal effects, thereby disrupting the crystalline structure of proteins, making them more prone to react with chemical reagents, resulting in better emulsifying properties. Furthermore, ultrasonication is a scalable, low-cost, and environmentally friendly technology which has little impact on the sensory of foods [15,16]. However, data concerning the comparative analysis of these different composite modification types remain limited.

Palm (*Elaeis guineensis* Jacq) oil has the largest production, consumption, and international trade volume in the world. Palm kernel cake is the main byproduct of palm oil processing, with an annual yield of approximately 430 million tons [17]. Palm kernel cake is rich in protein (23 g/100 g), of which glutelin-1 accounts for 24 g/100 g of palm kernel cake protein [18]. Palm kernel cake glutelin-1 (PKCG-1) exhibits high digestibility, good solubility under acidic conditions, and considerable foaming ability, together with antioxidant, antihypertensive, antibacterial, and iron-fortification activities [19,20,21,22]. More importantly, PKCG-1 has shown an emulsifying ability (91.03 m^2^/g) and emulsion stability (79.77%) [23] higher than that of soybean protein but lower than sugar esters and polysorbate-80 [23,24]. These findings indicated that PKCG-1 can be used as a safe and novel emulsifier, contingent upon the enhancement of its intrinsic emulsifying functionality. While limited enzymatic hydrolysis has been used to enhance the emulsifying properties of PKCG-1 [25], data regarding the effects of composite methods remains scare.

Accordingly, we hypothesized that both ultrasonication-assisted gallic acid binding or arabinose glycosylation can improve the emulsifying properties of PKCG-1. To test this hypothesis and advance the utilization of palm kernel cake, this study conducted a comparative analysis of the effects and underlying mechanisms of ultrasonication-assisted gallic acid binding and arabinose-glycosylation on the emulsifying properties of PKCG-1. This work offers guidance significance for expanding the applications of PKCG-1 and providing a foundation for the rational design of high-value emulsifiers from agro-industrial residues.

## 2. Results and Discussion

### 2.1. Substitution Degree of PKCG-1-UA and PKCG-1-UGA

The extraction rate of PKCG-1 was 8.85 ± 0.24 g/100 g of palm kernel cake, with a prior study obtaining a similar result (9.02 g/100 g) [23]. The degree of glycosylation of PKCG-1-UA and the substitution degree of PKCG-1-UGA were 11.76% ± 0.37% and 8.40% ± 0.19%, respectively, confirming the conjugation of PKCG-1 with arabinose or gallic acid after ultrasonication-assisted gallic acid-binding or arabinose-glycosylation, respectively. Importantly, the glycosylation degree of PKCG-1-UA was higher than that of pea protein isolate–arabinose [12], attributed to the ultrasonication assisted effect. Ultrasonication induces cavitation, microstreaming, and localized shear forces that partially unfold protein structures, exposing amino and hydroxyl groups in PKCG-1, thereby improving the glycosylation degree [16].

### 2.2. Structural Characteristics

#### 2.2.1. SDS-PAGE

**Figure 1:**
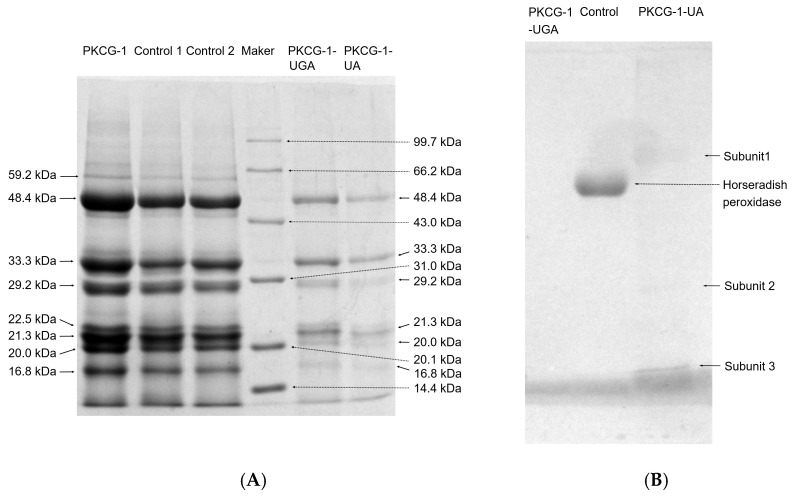
(**A**) SDS-polyacrylamide gel electrophoresis diagrams of PKCG-1, PKCG-1-UA, and PKCG-1-UGA that utilizing a 12% separating gel and 7.5% stacking gel. PKCG-1, oil palm kernel cake glutelin-1; PKCG-1-UA, palm kernel cake glutelin-1 modified via ultrasonication-assisted glycosylation with arabinose; and PKCG-1-UGA, palm kernel cake glutelin-1 modified via ultrasonication-assisted gallic acid grafting. Control 1: PKCG-1 modified by gallic acid-grafting without gallic acid; and Control 2: PKCG-1 modified by glycosylation without arabinose. Protein molecular weight markers included rabbit phosphorylase *b* (97.4 kDa), bovine serum albumin (66.2 kDa), rabbit actin (43.0 kDa), carbonic anhydrase (31.0 kDa), trypsin inhibitor (20.1 kDa), and egg white lysozyme (14.4 kDa). (**B**) SDS-polyacrylamide gel electrophoresis diagrams of PKCG-1-UA and PKCG-1-UGA stained using a commercially available glycoprotein staining kit with horseradish peroxidase as positive control.

The SDS-PAGE analysis showed that PKCG-1 contained nine main subunits with molecular weight in the range of 16.8–59.2 kDa (Figure 1A), which aligned with the findings of Zheng et al. [25]. Alternatively, the subunit with a molecular weight of 59.2 kDa disappeared in PKCG-1-UGA; whereas there were only three main subunits (with molecular masses of 48.4, 33.3, and 21.3 kDa) that remained in PKCG-1-UA, revealing that these dual modifications, especially ultrasonication-assisted glycosylation, induced the degradation of PKCG-1 and lowered its molecular mass [6,12]. Prior studies have shown that ultrasonic (80 kHz and 400 W) and heating treatment (90 °C, 30 min) during glycosylation induced the degradation of millet protein and ovalbumin, respectively [16,26]. To ascertain whether the structural and emulsifying alterations in PKCG-1 are caused by modification with arabinose and gallic acid, or by the conditions applied for glycosylation (pH 2.5 for 12 h; pH 7 at 90 °C for 30 min; dialysis) and gallic acid grafting (pH 7.8, 30 °C for 6 h; dialysis), two control experiment were conducted: Control 1 (glycosylation performed in the absence of arabinose) and Control 2 (gallic acid grafting without gallic acid). As shown in Figure 1A, no statistically significant differences were observed in subunit composition or molecular weight distribution among PKCG-1, Control 1, and Control 2 (*p* > 0.05), indicating that the conditions applied for glycosylation and gallic acid grafting alone did not induce detectable changes in the subunits and molecular weight distribution of PKCG-1.

Figure 1B illustrates the SDS-PAGE gel of PKCG-1-UGA and PKCG-1-UA stained using a glycoprotein staining kit with horseradish peroxidase as a positive control. Under these conditions, glycoproteins exhibit distinct bands, whereas non-glycosylated proteins do not display detectable bands on the gel [12]. Horseradish peroxidase had a clear stand, confirming reports that it contains 16 g/100 g of glycoprotein [27]. Relatively three faint bands (Subunit 1, 2, and 3) were observed in PKCG-1-UA, indicating the formation of conjugates between PKCG-1-UA and arabinose. The relatively low brightness of the subunits indicates a lower content of glycoproteins in PKCG-1-UGA, corresponding to the relatively low degree of glycosylation (11.76% ± 0.37%). In contrast, no bands were detected in PKCG-1-UGA, suggesting that neither PKCG-1 nor PKCG-1-UGA contained glycosyl groups.

#### 2.2.2. Fluorescence Spectra

**Figure 2:**
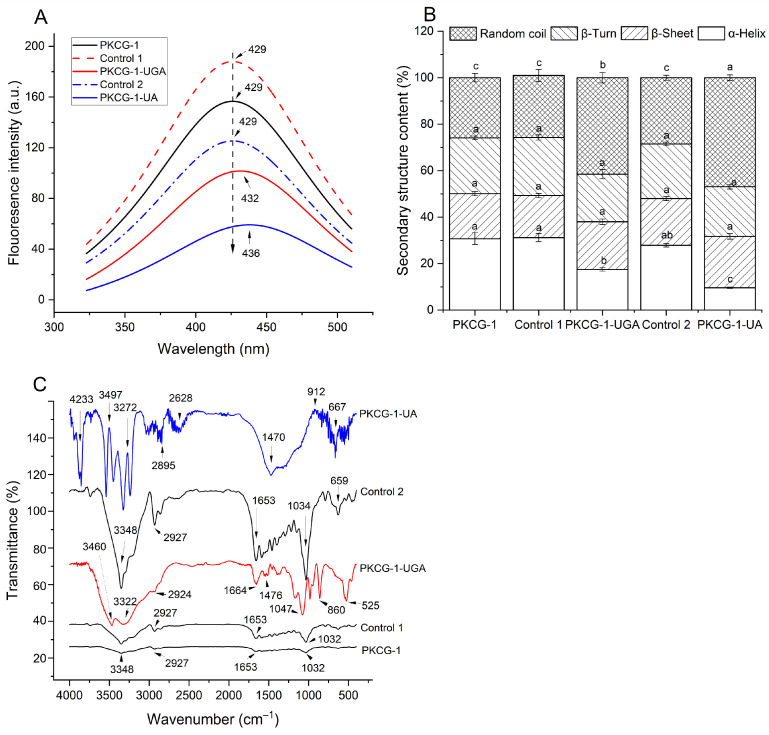
Influences of ultrasonication-assisted gallic acid grafting and arabinose glycosylation on the structural properties of PKCG-1: (**A**) endogenous fluorescence spectra; (**B**) secondary structure composition; and (**C**) Fourier-transform infrared spectroscopy of PKCG-1, PKCG-1-UA, and PKCG-1-UGA. Control 1: PKCG-1 modified by gallic acid-grafting in the absence of gallic acid; and Control 2: PKCG-1 modified by glycosylation without arabinose. Different lowercase letters (a–c) above bars indicate significant differences (*p* < 0.05).

As shown in Figure 2A, PKCG-1, Control 1, and Control 2 all exhibited a sharp peak at 429 nm (corresponding to the fluorescence adsorption of Ser and Trp) [28]. An obvious difference was found between the fluorescence spectra of PKCG-1, PKCG-1-UGA, and PKCG-1-UA. The sharp peak at 429 nm in the spectrum of PKCG-1 shifted to 432 and 436 nm in the spectra of PKCG-1-UGA and PKCG-1-UA, respectively. Moreover, the fluorescence intensity of PKCG-1-UGA and PKCG-1-UA was lower than that of PKCG-1, indicating that ultrasonication-assisted gallic acid-binding or arabinose-glycosylation caused the arrangement of hydrophobic amino acids in PKCG-1 [26]. Ultrasonication, glycosylation and gallic acid binding caused the degradation of PKCG-1′s subunits (Figure 1) and altered the spatial structure, resulting in the arrangement of hydrophobic groups and different fluorescence spectra [29]. These findings suggested that changes in intrinsic florescence spectra of PKCG-1 were attributed to arabinose or gallic acid conjugation, rather than to the conditions employed during glycosylation and gallic acid grafting.

#### 2.2.3. Circular Dichroism Spectrum Analysis

As illustrated in Figure 2B, ultrasonication-assisted gallic acid binding and glycosylation remarkably altered the secondary structure of PKCG-1 (Figure 2B). The α-helix (a rigid structure) content of PKCG-1 obviously decreased and random coil (a loose structure) content increased, evidencing that these dual modifications increased the flexibility of PKCG-1. Cavitation, microstreaming effects, heating, glycosylation or gallic acid-binding caused broken of the spatial structure PKCG-1 (Figure 1), resulting in the breakage of chemical bonds, thereby decreasing α-helix content. Furthermore, the random coil content of PKCG-1-UA was higher than that of PKCG-1-UGA, perhaps because the reaction conditions during gallic acid-binding were milder than that during glycosylation. Although previous studies have found that phenol binding can increase the β-sheet content of proteins [3], there was no statistical difference in β-sheet content between PKCG-1 and PKCG-1-UGA (*p* > 0.05). In contrast, there was no statistically significant differences in the secondary structural fractions (α-helix, random coil, β-sheet, and β-turn) among PKCG-1, Control 1, and Control 2 (*p* > 0.05). Although Control 2 exhibited a higher random coil content than PKCG-1, potentially attributed to the thermal denaturation induced by heating treatment (90 °C for 30 min) during glycosylation, this difference remained statistically insignificant (*p* > 0.05). A looser structure was conducive for PKCG-1-UGA and PKCG-1-UA to exhibit better interface properties [28].

#### 2.2.4. FT-IR Analysis

FT-IR spectra revealed distinct structural differences between PKCG-1, PKCG-1-UGA, and PKCG-1-UA, consistent with the modification by gallic acid and arabinose, respectively (Figure 2C). Specifically, the broad O–H/N–H stretching band centered at 3348 cm^−1^ in PKCG-1 shifted to lower wavenumbers—3322 cm^−1^ in PKCG-1-UGA and 3272 cm^−1^ in PKCG-1-UA—suggesting altered hydrogen-bonding environments due to conjugation. Concurrently, new absorption bands emerged at 3460 cm^−1^ (PKCG-1-UGA) and 3307 cm^−1^ (PKCG-1-UA), attributable to newly formed N–H or O–H stretches associated with grafting-induced conformational changes. Additionally, the amide I band, originally observed at 1653 cm^−1^ (C=O stretching), shifted to 1664 cm^−1^ in PKCG-1-UGA and to 1470 cm^−1^ in PKCG-1-UA. The former shift reflects the subtle perturbation of the peptide backbone, whereas the latter—falling within the aromatic C=C bending region—supports the incorporation of the gallic acid phenolic ring. These spectral changes collectively indicate that ultrasonication-assisted conjugation modified the secondary structure and hydrogen-bonding network within the amide I domain [26].

Further evidence for gallic acid binding was observed in PKCG-1-UGA: a blue shift of the C–O stretching vibration from 1032 to 1074 cm^−1^, along with newly resolved peaks at 900 cm^−1^ (C–C bending), 1001 cm^−1^ (aromatic C–H in-plane bending), and 1301 cm^−1^ (benzene ring skeletal vibration) [2,30], all characteristic of galloyl moieties [15]. In contrast, PKCG-1-UA exhibited additional absorptions at 4233 cm^−1^ (N–H stretching, overlapped with overtone), 3497 cm^−1^ (O–H stretching), 2628 cm^−1^ (C=O stretching, possibly from hemiketal or hydrated carbonyl), 912 cm^−1^ (C–O–C glycosidic bond stretching), and 667 cm^−1^ (C–O–H bending), corroborating successful arabinosylation [5,26]. Notably, the appearance of the 912 cm^–1^ band strongly supports linkage between the amino groups of PKCG-1 and the anomeric carbon of arabinose [13]. However, determining the more specific binding sites for PKCG–1-gallic acid conjugates requires further investigation.

In addition, there was no significant difference between the spectra of PKCG-1 and Control 1. A new absorption peak at 659 cm^−1^ appeared in the spectrum of Control 2, ascribed to C–O–H bending vibrations caused by thermal treatment during glycosylation [26]; however, no other spectral deviations were detected between PKCG-1 and Control 2.

Overall, the results in Figure 1 and Figure 2 revealed that the structural modifications observed in PKCG-1 were primarily caused by ultrasonication-assisted arabinose or gallic acid conjugation, not by the conditions employed during glycosylation or gallic acid grafting.

#### 2.2.5. Amino Acid Composition Analysis

As shown in Table 1, PKCG-1 is rich in glutamic acid and aspartic acid, followed by Leu, Tyr, and Lys, which was consistent with findings reported by Zarei et al. [21]. Compared to PKCG-1, both PKCG-1-UGA and PKCG-1-UA exhibited a similar amino acid profile, with a slight difference. Specifically, the contents of asparagine, lysine, and glutamic acid in PKCG-1-UA were lower than that of PKCG-1 (*p* < 0.05), indicating conjugation of these residues and arabinose during glycosylation [13]. The *ɛ*-NH_2_ of lysine and asparagine can undergo nucleophilic addition reaction with the carbonyl group (C=O) of arabinose to form an unstable Schiff base intermediate [12]. Furthermore, a prior study demonstrated that the Millard reaction can cause the conversion of glutamic acid to glutamine, resulting in a lower content of glutamic acid [5]. In contrast, the tyrosine, valine, proline, and phenylalanine contents of PKCG-1-UGA were lower than that of PKCG-1 (*p* < 0.05), implying involvement of these hydrophobic amino acid residues in conjugation with gallic acid. The non-polar aromatic rings of gallic acid preferentially associate with hydrophobic amino acid residues of protein [2].

Overall, the results in Figure 1 and Figure 2 revealed that the grafting of gallic acid and arabinose on PKCG-1 and ultrasonication-assisted gallic acid binding or arabinose glycosylation both decreased the molecular mass of PKCG-1 and increased its structural flexibility. However, more direct molecular confirmation techniques (e.g., nuclear magnetic resonance, liquid chromatography-mass spectrometry, and amino acid modification mapping) should be performed in further work.

### 2.3. Surface Hydrophobicity Analysis

**Figure 3:**
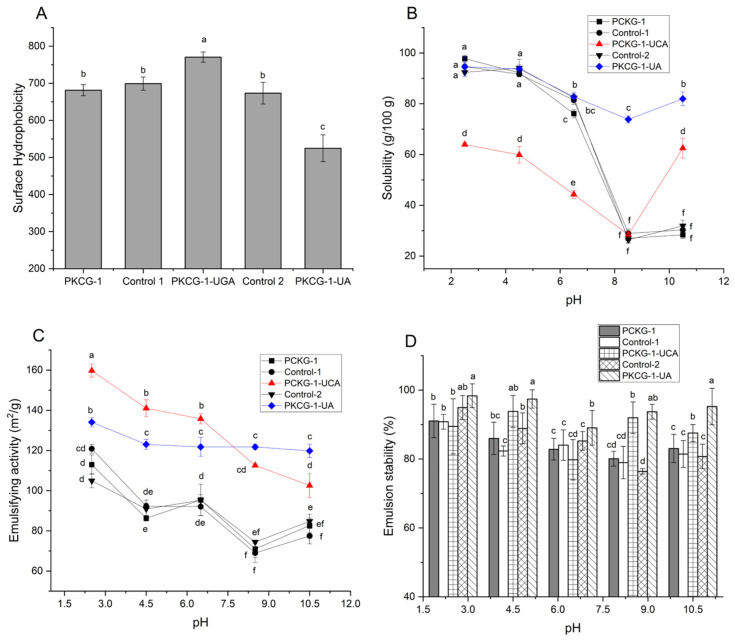
Surface hydrophobicity (**A**), solubility (**B**), emulsifying activity (**C**), and emulsion stability (**D**) of PKCG-1, PKCG-1-UA, and PKCG-1-UGA with in a pH range of 2 to 10. Control 1: PKCG-1 modified by gallic acid-grafting without gallic acid; Control 2: PKCG-1 modified by glycosylation without arabinose. Different lowercase letters (a–f) on the bars or data points indicate statically significant difference (*p* < 0.05).

The surface hydrophobicity of PKCG-1 was enhanced by ultrasonication-assisted gallic acid-grafting (Figure 3A), ascribed to the higher hydrophobicity of gallic acid [31]. Moreover, ultrasonication-assisted gallic acid binding caused degradation of subunits in PKCG-1 and increased random coil content (Figure 1 and Figure 2B), inducing the rearrangement of hydrophobic groups in PKCG-1 and enhancing its hydrophobicity [6]. Additionally, ultrasound can cause aggregation of protein–polyphenol conjugates and lower their surface hydrophobicity [15]. In contrast, PKCG-1-UA had lower hydrophobicity than PKCG-1 (*p* < 0.05). Conjugate of arabinose can significantly enhance the hydrophilicity of PKCG-1 [12] and consequently decrease its surface hydrophobicity. Additionally, the surface hydrophobicity of Control 1 and Control 2 did not differ significantly from that of PKCG-1, indicating that the conditions applied for glycosylation and gallic acid grafting did not substantially alter the hydrophobicity. The reason for this phenomenon was that these conditions had no statical impact on the structure of PKCG-1 (Figure 1 and Figure 2).

### 2.4. Solubility Under Different pH Conditions

The solubility of proteins is crucial for their ability to rapidly disperse in aqueous solution and absorb at water–oil interfaces [32]. As shown in Figure 3B, PKCG-1, PKCG-1-UGA, and PKCG-1-UA exhibited a solubility curve that decreased as pH increased from 2.5 to 10.5, with the lowest solubility at pH 8.5, which was consistent with the results of Chee et al. [20]. Moreover, these findings indicated that the isoelectric point of these PKCG-1s is near pH 8.5. Since PKCG-1 is rich in acidic amino acids (glutamic acid and aspartic acid) [25], it showed superior solubility under acidic conditions and an isoelectric point near pH 8.5. At the isoelectric point, the net charge of proteins is zero and the electrostatic repulsion between them is lowest, leading to aggregation and sediment [31].

Importantly, the solubility of PKCG-1-UA is higher than that of PKCG-1 at pH 6.5–10.5 (*p* < 0.05), predominantly ascribed to the introduced arabinose, which possesses multiple hydrophilic hydroxyl groups [14]. Moreover, following ultrasonication-assisted glycosylation, the molecular mass of PKCG-1 decreased (Figure 1) and its structure became looser (Figure 2B), which was conducive to exposing more hydrophilic groups and increasing the affinity of PKCG-1 to water [25]. Conversely, PKCG-1-UGA showed a lower solubility than that of PKCG-1 at pH 2.5–6.5, ascribed to the poor hydrophilicity of gallic acid [33]. Moreover, both Control 1 and Control 2 displayed a similar solubility profile relative to PKCG-1 (Figure 3B), suggesting that the conditions applied during glycosylation or gallic acid grafting did not have any obvious effect on the solubility of PKCG-1. Coincidentally, the solubility of yeast protein was also decreased by polyphenol-binding [6].

### 2.5. Emulsifying Capacity and Emulsion Stability

**Figure 4:**
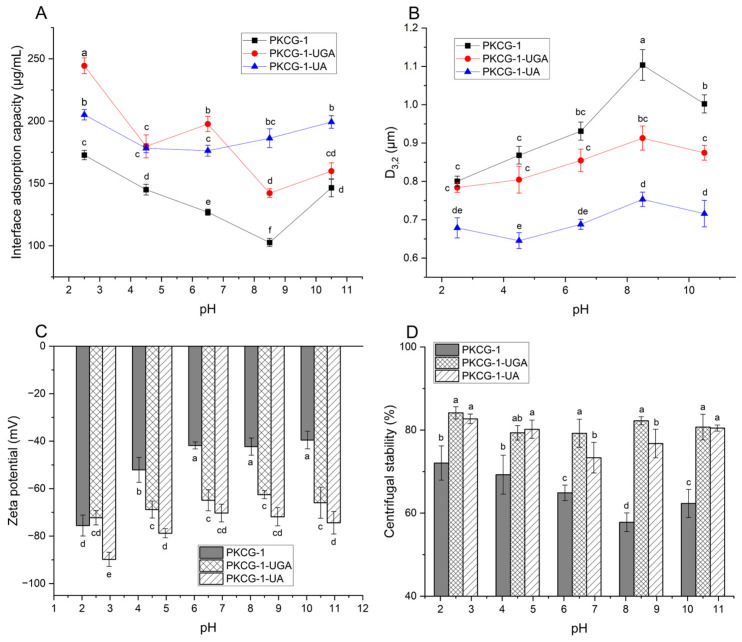
(**A**) Interfacial adsorption capacity of PKCG-1, PKCG-1-UA, and PKCG-1-UGA; (**B**) the particle size of droplets; (**C**) zeta potential, and (**D**) centrifugal stability of the emulsions stabilized by PKCG-1, PKCG-1-UA, and PKCG-1-UGA at different pH values. Different lowercase letters (a–f) on bars or data points denote statistically significant differences (*p* < 0.05).

Compared to PKCG-1, a higher emulsifying capacity and emulsion stability were observed in both PKCG-1-UGA and PKCG-1-UA (Figure 3C,D), evidencing that ultrasonication-assisted gallic acid binding or arabinose glycosylation enhanced the emulsifying properties of PKCG-1. Ultrasonication-assisted gallic acid-binding or arabinose-glycosylation enhanced the hydrophobicity and solubility of PKCG-1 (Figure 3A,B), increasing the affinity of PKCG-1 to oil and water, respectively, thereby improving its adsorption capacity at the water–oil interface (Figure 4A) and emulsifying activity. Moreover, the looser structure and lower molecular weight of PKCG-1-UGA and PKCG-1-UA (Figure 1 and Figure 2B) were helpful for adsorption at the interface. Furthermore, the hydroxyl groups in the introduced gallic acid and arabinose were proven to reduce free energy in the emulsion, which was helpful for forming an interfacial film on the surface of oil droplets [2,13], resulting in a stronger repulsive force between the droplets and better emulsion stability. In addition, PKCG-1-UGA showed a higher emulsifying ability than PKCG-1-UA at pH 2.5 and 8.5, mainly ascribed to the higher surface hydrophobicity of PKCG-1-UGA (Figure 3A). Increased surface hydrophobicity remarkably enhanced the affinity of proteins to oils, which is crucial for emulsifying ability [7]. A prior study showed that ultrasonication can cause aggregation of protein–polyphenol conjugates at the water–oil interface, thereby increasing emulsifying capacity [16]. Alternatively, PKCG-1-UA exhibited a better emulsion stability than PKCG-1-UA at pH 2.5, 8.5, and 10.5 (*p* < 0.05). PKCG-1-UA had a higher random coil content (Figure 2B), lower mass (Figure 1A), and superior solubility (Figure 3B) than PKCG-1-UGA, which was conducive to forming a resilient interfacial film surrounding droplets and increasing the stability of emulsions [1,26]. These findings indicated that ultrasonication-assisted gallic acid binding was the most effective method for improving the emulsifying ability of PKCG-1, while ultrasonication-assisted arabinose glycosylation was best at improving its emulsion stability.

Apart from that, PKCG-1, PKCG-1-UGA, and PKCG-1-UA exhibited emulsifying ability and emulsion stability profiles that decreased as pH increased from 2.5 to 8.5, with the lowest values at pH 8.5, which was consistent with their solubility curves against pH (Figure 3B), confirming the report that solubility profoundly affects the emulsifying properties of proteins [7]. PKCG-1, an acid-soluble protein, had the highest solubility at pH 2.5; increasing pH decreased its solubility (Figure 3B). A decrease in solubility can reduce the ability of proteins to adsorb at the water–oil interface and decrease the repulsive force between droplets and reducing the thickness of the electric double layer, resulting in poor emulsifying activity and emulsion stability [34]. At pH 8.5 (near the isoelectric point), PKCG-1s had the lowest net charge and poorest ability to prevent the aggregation of droplets [3], resulting in poor emulsion stability. Additionally, the emulsifying activity (159.74 and 134.08 m^2^/g, respectively) and emulsion stability (89.36% and 98.03%, respectively) of PKCG-1-UGA and PKCG-1-UA were nearly equal to those of sugar esters [24], indicating their potential as efficient emulsifiers.

The emulsifying activity of Control 1 and Control 2 did not statically differ from that of PKCG-1 over the pH range of 2.5–10.5 (*p* > 0.05; Figure 3C). As pH increased from 2.5 to 4.5, Control 1 exhibited a comparable emulsion stability profile to PKCG-1. Although Control 2 showed a higher emulsion stability than PKCG-1 at pH 2.5 and 4.5, these differences remained statistically insignificant (*p* > 0.05). Collectively, these results indicate that the applied conditions during glycosylation and gallic acid grafting did not induce any significant changes in the emulsifying properties of PKCG-1, ascribed to the subtle impact of the treatments on the structure (Figure 1 and Figure 2), hydrophobicity, and solubility (Figure 3A,B). Therefore, the study of the mechanism by which the conditions applied for glycosylation and gallic acid grafting affect the emulsifying properties of PKCG-1 is unnecessary.

### 2.6. Underlying Mechanism Insights

#### 2.6.1. Interface Sorption Capacity

PKCG-1, PKCG-1-UGA and PKCG-1-UA all exhibited a profile of interface adsorption against pH (Figure 4A) similar to their emulsifying activity curves (Figure 3C), evidencing that interface adsorption capacity plays an important role in the emulsifying property of proteins [34]. PKCG-1-UA and PKCG-1-UGA exhibited a higher interface adsorption capacity than PKCG-1 at pH 2.5–8.5 (*p* < 0.05). The smaller molecular mass (Figure 1) and higher random coil content (Figure 2B), and higher solubility and hydrophobicity (Figure 3A,B), were conducive for PKCG-1-UA and PKCG-1-UGA to adsorb at water–oil interface and form a resilient interfacial film [2]. Moreover, these PKCG-1s exhibited a decreasing interface sorption capacity as pH increased from 2.5 to 10.5, showing the lowest capacity at pH 8.5. At the isoelectric point, PKCG-1s had the lowest net charge and poorest affinity to water and oil [26], exhibiting low interface adsorption capacity. Additionally, PKCG-1, PKCG-1-UA, and PKCG-1-UGA exhibited higher interface adsorption capacities (172.76–244.41 μg/mL) than soy protein (95.33 μg/mL) [24], which was the main reason for their superior emulsifying ability.

#### 2.6.2. Particle Size of the PKCG-1-Based Emulsions

As illustrated in Figure 4B, ultrasonication-assisted gallic acid-binding or arabinose-glycosylation decreased the particle size (D_3,2_) of PKCG-1-stabilized emulsion (*p* < 0.05), which was consistent with the superior emulsion stability of PKCG-1-UA and PKCG-1-UGA (Figure 3D). Ultrasonication-assisted gallic acid-binding or arabinose-glycosylation made the structure of PKCG-1 become looser (Figure 2B) and increased its solubility and hydrophobicity, respectively, enhancing its affinity to water and oils, thereby increasing the repulsive force between droplets and thickening the double layer [34], leading to a smaller emulsion droplet size. The PKCG-1-UA stabilized emulsion had a smaller particle size than that of PKCG-1-UGA, contributing to its better emulsion stability. Ultrasonication can cause aggregation of protein–polyphenol conjugates, resulting in the larger droplet size of the PKCG-1-UGA stabilized emulsion.

Furthermore, the emulsions stabilized by PKCG-1, PKCG-1-UGA, and PKCG-1-UA exhibited an increasing particle size as pH increased from 2.5 to 8.5, with the largest D_3,2_ at pH 8.5. The charge in the double layer covering droplets was lowest near the isoelectric point; consequently, the repulsive force between droplets decreased, resulting in emulsion aggregation and a larger size [1]. Away from this point, the net charge in proteins increased and the repulsive force between droplets was enhanced, increasing the distance between them and increased leading to a decrease in particle size [6].

#### 2.6.3. Zeta-Potential Profiles

As shown in Figure 4C, PKCG-1-UA- and PKCG-1-UGA-based emulsions showed higher absolute zeta-potential than PKCG-1 at pH 4.5–10.5 (*p* < 0.05) (Figure 4C), contributing to their better emulsion stability (Figure 3D). After ultrasonication-assisted gallic acid binding or arabinose glycosylation, the structure of PKCG-1 became looser, and its solubility, hydrophilicity, and interface adsorption capacity were increased (Figure 3A,B and Figure 4A), elevating the quantity of electrostatic charge on the slipping plane of the double layer and resulting in a higher absolute zeta-potential [9]. The zeta-potential of OPKCG-1-UA was higher than that of PKCG-1-UGA at pH 2.5, 4.5, and 10.5, ascribed to its better emulsion stability (Figure 3D). A previous study found that arabinose glycosylation can enhanced the zeta-potential of emulsions through enhancing the interaction between emulsifiers and water/oils and increasing the viscosity of the emulsions [13].

Moreover, the zeta-potential of the PKCG-1-based emulsions was lowest at pH 8.5 and increased when pH moved away from this point. The net charge of PKCG-1s was lowest near the isoelectric point, reducing the repulsive force between droplets [11], thereby decreasing the zeta-potential. Away from the isoelectric point, the dissociation of PKCG-1s was enhanced, which increased the ionic layer of the slipping plane and the thickness of the double electric layer, resulting in a higher absolute zeta-potential [26]. A similar trend was obtained by Ravindran et al. [34].

#### 2.6.4. Centrifugal Stability of the Emulsions

**Figure 5:**
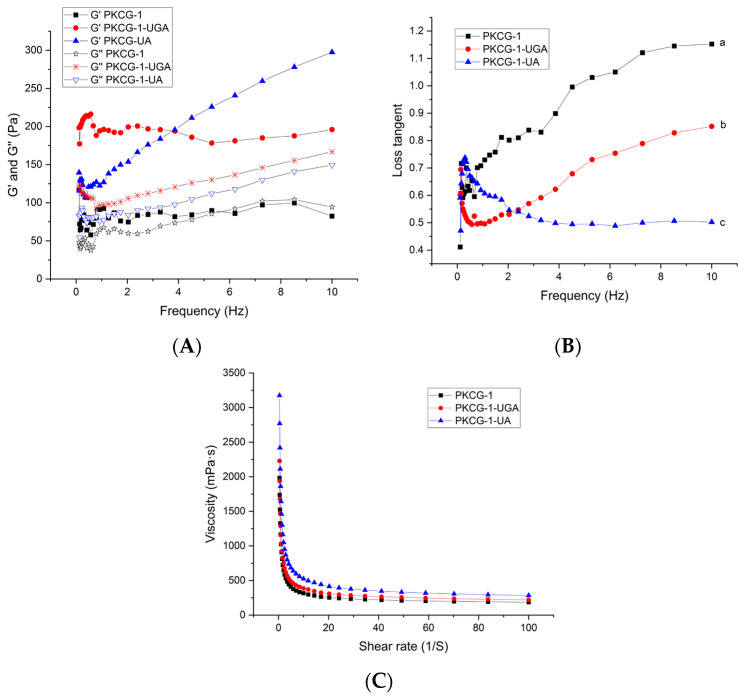
Rheological characteristics of the PKCG-1-, PKCG-1-UA-, and PKCG-1-UGA-based emulsions. (**A**) Energy storage modulus (G′) and loss modulus (G″); and (**B**) loss factor of the emulsions at various frequencies; and (**C**) viscosity at different shear rates. Different lowercase letters (a–c) near lines indicate statistically significant differences (*p* < 0.05).

The PKCG-1-UA- and PKCG-1-UGA-based emulsions showed better centrifugal stability at pH 2.5–10.5 than PKCG-1 (*p* < 0.05), ascribed to the higher random coil content (Figure 2B) and interface adsorption capacity of PKCG-1-UA and PKCG-1-UGA (Figure 4A), together with the lower droplet size and loss tangent and higher zeta-potential in the emulsions (Figure 4B,C and Figure 5B). An increase in structural flexibility, interface adsorption capacity, and viscosity enhanced the toughness of the emulsion film covering the droplets [15], while a lower loss tangent confirmed the formation of a stable emulsion film, which was crucial to the centrifugal stability of the emulsions [1]. Additionally, the PKCG-1-based emulsions showed better centrifugal stability (72.05–84.14%) than that of soy protein-based emulsions (54.91%) [9], corresponding to the considerable emulsion stability of PKCG-1 (Figure 3D).

Coincidentally, the centrifugal stability of the PKCG-1- and PKCG-1-UA-based emulsions were lowest at pH 8.5, corresponding to their lowest emulsion stability. Near the isoelectric point, the electrostatic repulsion and physical barrier forces between the droplets were poor, thereby reducing the resistance of emulsions to centrifugal force [34]. Alternatively, the centrifugal stability of the PKCG-1-UGA-based emulsion did not remarkably alter when pH increased from 2.5 to 10.5 (*p* > 0.05), revealing that ultrasonication-assisted glycosylation reduced the sensitivity of the centrifugal stability of the PKCG-1-based emulsion. Yang et al. [14] found that arabinose-glycosylation enhanced the centrifugal stability of coconut protein-stabilized emulsions. However, the influence of ultrasonication, glycosylation, and gallic acid grafting on the stability of the PKCG-1-based emulsion during storage and processing requires further work.

#### 2.6.5. Rheological Characteristics

A decrease in the loss tangent (tan δ = G″/G′) suggested a decreased viscous dominance and correspondingly increased structural integrity in the emulsions [9]. As illustrated in Figure 5A,B, the PKCG-1-based emulsion exhibited G″ > G′ and a high loss tangent (>1) at a frequency above 5 Hz, corroborating its relatively poor emulsion stability (Figure 3D). In contrast, the PKCG-1-UA- and PKCG-1-UGA-stabilized emulsions exhibited a lower tan δ (<1) within a frequency range of 0.1 to 10 Hz, demonstrating that both ultrasonication-assisted glycosylation and gallic acid biding can improve the emulsion stability of PKCG-1 via reducing the loss tangent. Ultrasonication-assisted glycosylation and gallic acid biding markedly increased the interfacial adsorption capacity of PKCG-1 (Figure 4A), concurrently reducing droplet size and elevating zeta potential (Figure 4B,C), which was conducive to the formation of a robust, viscoelastic interfacial film and a concomitant reduction in the loss tangent [1].

The PKCG-1-, PKCG-1-UA-, and PKCG-1-UGA-based emulsions were non-Newtonian fluids, for they all exhibited characteristic shear-thinning behavior (Figure 5C) [15]. Furthermore, the viscosity of the PKCG-1-UA-based emulsion was higher than that of the PKCG-1-based emulsion, which was one reason for the superior emulsion stability of PKCG-1-UA (Figure 3D). Wei et al. [13] and Xing et al. [1] found that arabinose and phenolic acid conjugation enhanced the stability of chickpea and egg white protein-based emulsions, respectively, primarily through viscosity-mediated reinforcement. Additionally, the low loss tangent and relative high viscosity of PKCG-1-, PCKG-1-UA-, and PKCG-1-UGA-based emulsions indicated their potential application as emulsion gels.

#### 2.6.6. Cryogenic Scanning Electron Microscopy Profiles

**Figure 6:**
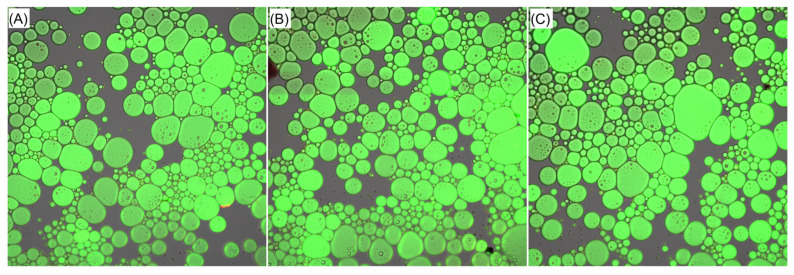
Cryogenic laser confocal scanning electron microscopy of the emulsions based on PKCG-1 (**A**), PKCG-1-UGA (**B**), and PKCG-1-UA (**C**) at a magnification of 40× with a scale bar of 50 μm. Emulsions were stained with Nile red and Nile Blue A Sulfate.

The cryogenic scanning electron microscopy images are shown in Figure 6A–C. Numerous droplets of different sizes were evenly distributed throughout the emulsions stabilized using PKCG-1, PKCG-1-UGA, and PKCG-1-UA, consistent with their considerable emulsifying activity (91.03 to 159.74 m^2^/g) and emulsion stability (79.77–98.36%). No real visual difference can be deduced between the emulsions stabilized with PKCG-1 and PKCG-1-UA. However, according to visual estimation, compared to PKCG-1, the number of spherical emulsion droplets was larger, corresponding to the better emulsifying activity and emulsion stability of PKCG-1-UGA. Moreover, the increased red spherical droplets in Figure 5B,C suggested that ultrasonication-assisted arabinose or gallic acid conjugation improved the ability of PKCG-1 to form a resilient emulsion film [2]. This phenomenon was attributed to the higher structural flexibility, lower molecular mass, and higher interface adsorption ability of PKCG-1-UGA and PKCG-1-UA, enhancing their capacity to reduce droplet size, zeta-potential, and the loss tangent of emulsions (Figure 1, Figure 2B and Figure 5B). Similar findings were obtained by Chen et al. [12], Yang et al. [14], and Abou-Elsoud et al. [15].

## 3. Conclusions

The results of this study revealed that ultrasonication-assisted gallic acid-binding or arabinose-glycosylation both decreased the molecular mass of PKCG-1, increased its random coil content, and enhanced its emulsifying activity and emulsion stability (*p* < 0.05). Specifically, ultrasonication-assisted gallic acid binding was most effective in improving the emulsifying ability of PKCG-1 (from 91.03 to 159.74 m^2^/g) by increasing its hydrophobicity, random coil content, and interfacial adsorption capacity, while reducing the loss tangent and augmenting the zeta-potential (from −39.55 to −65.96 mV). Moreover, ultrasonication-assisted arabinose glycosylation exhibited the best effect in enhancing the emulsion stability of PKCG-1 (from 79.77% to 98.36%), ascribed to increase in solubility (28.35 to 73.85 g/100 mL) and random coil content (25.9% to 46.9%), the enhancement of zeta-potential (−39.55 to −84.81 mV) and viscosity, and a reduction in the droplet size (1.10 to 0.64 μm) and loss factor of the emulsion. Furthermore, increasing pH value (from 2.5 to 8.5) decreased the solubility, emulsifying activity, and emulsion stability of PKCG-1. Notably, ultrasonication-assisted arabinose glycosylation attenuated the pH sensitivity of the emulsion stability. However, interpreting the changes in the emulsifying properties of PKCG-1 relative to the controls without the addition of arabinose and gallic acid requires further studies as does the more specific mechanisms of these dual modifications and the structure–function relationship and application domain of PKCG-1s. Finally, the effects and mechanisms of single ultrasonication and glycosylation with arabinose or gallic acid grafting on the emulsifying properties of PKCG-1, and the relationship between structural modification and gel behavior, require further investigation.

## 4. Materials and Methods

### 4.1. Materials

Palm kernel cake was obtained from Bigzhipo Oil Palm Processing Company, Haikou, China. Gallic acid, Nile Blue A Sulphate, 8-Anilino-1-Naphthalenesulfonic acid (ANS), disodium hydrogen phosphate, and other reagents were of analytical grade (purity > 97.5%) and purchased from Soliabo Reagents Co., Ltd., Tianjin, China. Glycine, Coomassie blue R-250, acrylamide, sodium dodecyl sulfate, and tetramethylethylenediamine were of electrophoretic grade and purchased from Yijun Co., Ltd., Beijing, China. Alcalase (1.0 × 10^4^ U/mg) and protein markers were purchased from Sinopharm Biochemical Institute, Nanjing, China.

### 4.2. Extraction of PKCG-1

Palm kernel cake was crushed and sieved with a 60-mesh sieve, and the obtained powder was deoiled with petroleum ether (1:20, *m/v*) in triplicate [23]. The defatted palm kernel cake was crushed and sieved with a 100-mesh sieve. To exclude the albumin, glutelin-1, and glutelin-2, the fine powder (30 g) was dispersed in 300 mL of phosphate buffer (0.2 mol/L, pH 7.8, containing 0.2 mol/L of NaCl and 10 μmol/L of β-mercaptoethanol). The extraction solution was stirred at 180 r/min and 35 °C using a YSAH-I thermostatic water bath oscillator (Xingyuan Star Science Instrument Factory, Shaoxing, China) for 150 min. After filtration through a filter paper, the residue was collected and mixed with 200 mL of acetic acid (50%, *v*/*v*) and shaken at 180 r/min and 35 °C using YSAH-I thermostatic water bath oscillator for 120 min. The mixture was filtered using a filter paper, and the filtrate was centrifuged at 12,000× *g* using a KH22R frozen high-speed centrifuge (Kaida Scientific Instrument Co., Ltd., Changsha, China) for 20 min. The supernatant was collected and dialyzed against deionized water (dH_2_O) employing PS213590 dialysis membranes with a 3500 Da molecular weight cutoff (MWCO) at 4 °C for 48 h. The dH_2_O was changed at 4 h intervals. Following lyophilization using an AFD150 lyophilizer (Shengke Vacuum Dry Instrument Factory, Nanjing, China), PKCG-1-1 was obtained. The extraction rate (g/100 g) was defined as the obtained PKCG-1 per weight of palm kernel cake.

### 4.3. Ultrasonication of PKCG-1

Based on a modified method of Liu et al. [35], PKCG-1 (5 g) was dispersed in 250 mL of 0.1 mol·L^−1^ sodium phosphate–citric acid buffer (pH 2.5) and homogenized using a HulaMixer™ vortex mixer (Thermo Fisher Scientific, Waltham, MA, USA) at 1200 rpm for 12 s. The resulting dispersion was subjected to ultrasonic treatment in a LanJ-L45 ultrasonic cleaner (inner tank size of 450 × 300 × 300 mm; Jialang Ultrasonic Electrical Appliance Co., Ltd., Guangzhou, China) operating at 80 kHz, 40 °C, and 400 W (power density of 8.45 W/mL) for 45 min. Subsequently, the treated dispersion was aliquoted into two equal portions for subsequent glycosylation and gallic acid grafting, respectively.

### 4.4. Glycosylation of the Ultrasonic-Treated PKCG-1

Following the method of Chen et al. [12] with some modifications, ultrasonicated PKCG-1 (80 mg) was dissolved in 200 mL of 0.1 mol·L^−1^ sodium phosphate–citric acid buffer (pH 2.5), followed by the addition of L-arabinose (40 mg). The mixture was vortexed thoroughly and incubated at 25 °C under continuous shaking (135 rpm) for 12 h. The reaction mixture was then adjusted to pH 7.0 with 1 mol·L^−1^ NaOH and heat-treated at 90 °C for 30 min to terminate enzymatic or non-enzymatic side reactions. After rapid cooling on ice, the solution was centrifuged at 10,000× *g* for 10 min at 4 °C. The supernatant was dialyzed against deionized water (dH_2_O) using a regenerated cellulose membrane (molecular weight cutoff: 3.5 kDa) at 4 °C for 48 h, with buffer exchanged every 4 h. Finally, the dialysate was lyophilized using an FD-1A-50 freeze dryer (Beijing Boyikang Laboratory Instruments Co., Ltd., Beijing, China) to yield palm kernel cake glutelin-1 modified via ultrasonication-assisted arabinose glycosylation (PKCG-1-UA). The yield (g per 100 g dry PKCG-1) was calculated as the mass of purified PKCG-1-UA obtained relative to the initial mass of dry PKCG-1. A control (Control 1) was performed at the same conditions without the addition of arabinose. The degree of glycosylation was quantified according to the o-phthaldialdehyde (OPA) assay protocol described by Spotti et al. [36].

### 4.5. Gallic Acid Grafting onto Ultrasonicated PKCG-1

Gallic acid grafting was performed on ultrasonicated PKCG-1 following a modified version of the method reported by Ribeiro et al. [6]. Briefly, 100 mL of the ultrasonicated PKCG-1 dispersion (prepared as described in Section 2.3) was mixed with gallic acid (1 g) and vortexed at 1200 rpm for 12 s using a SIT-II vortex oscillator (Xiwu Vortex Instrument Factory, Zhenjiang, China). The pH of the mixture was adjusted to 7.8 using 1 mol·L^−1^ NaOH, and the reaction was carried out under continuous agitation (115 rpm) at 30 °C for 6 h using a YSAH-I orbital shaker (Yueyang Sheng’an Instrument Co., Ltd., Yueyang, China). Upon completion, the reaction mixture was dialyzed against dH_2_O at 4 °C for 48 h (buffer exchanged every 4 h). The dialysate was subsequently lyophilized using the FD-1A-50 freeze dryer to afford palm kernel cake glutelin-1 modified via ultrasonication-assisted gallic acid grafting (PKCG-1-UGA). Yield was expressed as grams of PKCG-1-UGA obtained per 100 g of dry PKCG-1. A control (Control 2) was performed at the same conditions without the addition of gallic acid. The degree of gallic acid grafting was determined spectrophotometrically using the Folin–Ciocalteu assay [37].

### 4.6. Structural Characterization

#### 4.6.1. Sodium Dodecyl Sulfate Polyacrylamide Gel Electrophoresis (SDS-PAGE)

SDS-PAGE analysis was conducted using a Mini-G4 vertical electrophoresis system (Bio-Rad Laboratories, Shanghai, China) according to the Laemmli protocol [38]. Separating and stacking gels were prepared at final concentrations of 12% (*w*/*v*) and 7.5% (*w*/*v*), respectively; sample loading concentration was 1 mg·mL^−1^. Gels were stained overnight with Coomassie Brilliant Blue R-250 (1 g·100 mL^−1^) and destained with a solution containing 10% (*v*/*v*) phosphoric acid, 10% (*v*/*v*) glacial acetic acid, and 10% (*v*/*v*) ethanol for 8 h, with destaining solution refreshed every 30 min. Protein molecular weights were estimated using a standard protein ladder (Bio-Rad Precision Plus Protein Standards) comprising rabbit phosphorylase b (97.4 kDa), bovine serum albumin (66.2 kDa), rabbit actin (43.0 kDa), carbonic anhydrase (31.0 kDa), trypsin inhibitor (20.1 kDa), and egg white lysozyme (14.4 kDa), visualized and analyzed using a BOXF3 gel imaging system (Synene Co., Ltd., Tokyo, Japan). Subsequently, to identify the glycoprotein, the SDS-PAGE gel was stained using a commercially available glycoprotein staining kit (Zhongke Ruitai Biotechnology Co., Ltd., Beijing, China), with horseradish peroxidase serving as a positive control. According to the instructions in the manual, the loading concentration of the sample and control was 1.5 mg·mL^−1^, and the gel was immersed in 50 mL of 50% methanol (*v*/*v*) and shaken at 55 rpm for 30 min. After washing twice with 50 mL of ultrapure water, the gel was oxidized with 25 mL of oxidation reagent at 30 °C for 15 min, and stained using 25 mL of the glycoprotein staining solution at 55 rpm and 25 °C for 15 min [27].

#### 4.6.2. Intrinsic Fluorescence Spectra

Intrinsic fluorescence spectra were acquired for PKCG-1, PKCG-1-UA, and PKCG-1-UGA. Each sample (250 μg) was dissolved in 1 mL of 10 mmol·L^−1^ sodium phosphate–citric acid buffer (pH 6.0) [1]. Spectra were recorded using a Y-F100 fluorescence spectrophotometer (Iphisong Technology Co., Ltd., Tianjin, China) with excitation at 290 nm, emission scanning from 200 to 500 nm, and slit widths set to 5 nm for both excitation and emission.

#### 4.6.3. Circular Dichroism (CD) Spectroscopy

Far-UV circular dichroism spectra were collected for PKCG-1, PKCG-1-UA, and PKCG-1-UGA using a JASCO-1500 spectropolarimeter (JASCO Corporation, Tokyo, Japan) over a wavelength range of 190–260 nm, at a scan rate of 100 nm·min^−1^ and a path length of 0.1 cm [15]. Sample concentration was standardized to 100 μg·mL^−1^ in 10 mmol·L^−1^ sodium phosphate–citric acid buffer (pH 6.0). All measurements were performed in triplicate at 25 °C, and raw CD data were processed and deconvoluted using CDNN 2.1 software (Simple Spectra module) to estimate secondary structural composition.

#### 4.6.4. Fourier-Transformed Infrared (FT-IR) Spectroscopy

FT-IR spectra were acquired using a Nolay-20 spectrometer (Nolayda Technology Co., Ltd., Qingdao, China). Samples (1 mg) were mixed with dried KBr (150 mg), ground homogeneously in an agate mortar, and pressed into transparent pellets (1–2 mm thickness) under vacuum. Spectra were recorded in transmittance mode across the wavenumber range of 4000–400 cm^–1^ at a resolution of 4 cm^–1^ and with 32 co-added scans per spectrum [31].

#### 4.6.5. Amino Acid Composition

The amino acid compositions of PKCG-1, PKCG-1-UA, and PKCG-1-UGA were determined using an L-3100P amino acid automatic analyzer (Jiangnan Meihua Instrument Technology Co., Ltd., Wuxi, China), according to the protocol described by Chee et al. [20].

### 4.7. Emulsifying Properties and Related Functional Characteristics

#### 4.7.1. Surface Hydrophobicity

Surface hydrophobicity was determined according to the method of De la Cruz-Torres et al. [39], with minor modifications. Briefly, 20 μL of 8 mmol/L 8-anilino-1-naphthalenesulfonic acid (ANS) solution (pH 7.8) was added to 2 mL of PKCG-1s aqueous solutions at concentrations ranging from 10 to 600 μg/mL. Fluorescence intensity was measured using a FL6500 fluorescence spectrophotometer (PerkinElmer Inc., Waltham, MA, USA) with excitation at 390 nm, emission at 470 nm, and slit widths of 5 nm for both excitation and emission monochromators. Surface hydrophobicity was quantified as the initial slope of the fluorescence intensity versus the PKCG-1 concentration curve.

#### 4.7.2. pH-Dependent Solubility

PKCG-1s dispersions (2 g/100 mL in deionized water) were adjusted to pH 2.5, 4.5, 6.5, 8.5, and 10.5 using 1 mol/L HCl or NaOH [16]. The dispersions were equilibrated under continuous agitation (160 rpm) at 25 ± 1 °C for 30 min using an ASYH-2 orbital shaker. The dispersions were centrifuged at 3547× *g*, and the supernatant was pooled. The Bradford method [40] was used to determine the protein concentration. Solubility (%) was calculated according to Equation (1):

(1) $$Solubility(g/100g)=(C_{S}\times V_{S})/W_{M}\times 100$$

where *C_S_* (mg/mL) and *V_S_* (mL) denote the protein concentration and volume of the supernatant, respectively, and *W_M_* (g) represents the mass of PKCG-1s initially dispersed.

#### 4.7.3. Emulsifying Activity and Emulsion Stability

The emulsifying activity index (EAI) and emulsion stability index (ESI) were assessed using the turbidimetric method described by Pearce & Kinsella [41]. PKCG-1s solutions (4.5 g/100 mL) were adjusted to the same pH values as above. Each solution (3 mL) was mixed with soybean oil (1 mL) and homogenized at 20,000 rpm for 120 s using an HSD-I40 high-shear homogenizer (Lihu Homogenizing Instrument Co., Ltd., Wuxi, China). Immediately after homogenization, 40 μL of the freshly prepared emulsion was diluted into 2600 μL of 1 mg/mL sodium dodecyl sulfate (SDS) solution, and absorbance at 500 nm (*A*_0_) was recorded. A second absorbance measurement (*A_t_*) was taken after 10 min of static incubation at 25 °C. EAI (m^2^/g) and ESI (%) were calculated using Equations (2) and (3), respectively:

(2) $$Emulsifying\;activity\;(m^{2}/g)=(2.303\times 2\times A_{0}\times f)/(C\times \varphi \times 10,000)$$

(3) $$Emulsion\;stability\left(\%\right)=A_{t}/A_{0}\times 100$$

where 2 accounts for the two-phase interfacial area; 2.303 is ln(10); *f* = 50 is the dilution factor; *C* (g/mL) is the PKCG-1s concentration in the aqueous phase prior to emulsification; and *φ* = 0.25 is the volume fraction of soybean oil in the emulsion system.

### 4.8. Mechanistic Investigation

#### 4.8.1. Emulsion Preparation

Emulsions stabilized by PKCG-1, PKCG-1-UA, and PKCG-1-UGA were prepared across the pH range 2.5–10.5 following the protocol outlined in Section 4.7.3.

#### 4.8.2. Interface Sorption Ability

Interfacial adsorption capacity was evaluated based on the method of Xing et al. [1]. Briefly, 20 mL of each PKCG-1s-stabilized emulsion was centrifuged at 8460× *g* for 15 min at 4 °C. The upper oil layer was carefully removed, and the remaining aqueous phase was subjected to three consecutive centrifugation–decantation cycles to eliminate non-adsorbed (unbound) protein. The pooled aqueous phase was analyzed for residual protein content using the Bradford assay [40]. Interfacial adsorption capacity (%) was calculated as per Equation (4):

(4) $$Interface\;sorption\;ability={(C}_{0}-C_{f})/C_{0}\times 100\%$$

where *C*_0_ and *C_f_* are the initial and final protein concentrations in the aqueous phase, respectively.

#### 4.8.3. Particle Size Distribution and Zeta-Potential

Volume- and area-weighted mean particle size (D_3,2_) and zeta potential of PKCG-1s-stabilized emulsions were determined using a Malvern Panalytical Zetasizer Pro instrument (Malvern Panalytical Ltd., Malvern, UK), following the procedure of Nobakht-Nia et al. [32]. Prior to analysis, emulsions were diluted 100-fold with deionized water. The diluted sample was gently transferred into a disposable U-shaped quartz cuvette, avoiding bubble formation. Measurements were conducted at 25 °C with automatic viscosity and refractive index correction applied.

#### 4.8.4. Centrifugal Stability

Centrifugal stability was assessed according to Xing et al. [1]. Absorbance at 500 nm (*A*_0_) of 10 mL of freshly prepared emulsion was first recorded. Samples were then centrifuged at 5000× *g* for 20 min at 4 °C. Thereafter, 10 μL of the sedimented bottom phase was withdrawn and diluted with 3 mL of deionized water; the resulting suspension’s absorbance (*A_t_*) was measured at 500 nm. Centrifugal stability (%) was calculated using Equation (5):

(5) $$Centrifugal\;stability={(A}_{0}-A_{t})/A_{0}\times 100\%$$

#### 4.8.5. Rheological Behavior

According to the method of Ballabio et al. [24], the rheological properties of PKCG-1s-stabilized emulsions were characterized using a DHR-3 rotational rheometer (TA Instruments, formerly Baosheng Industrial Development Co., Ltd., Shanghai, China), equipped with a 50 mm parallel-plate geometry (PP50) and a Peltier temperature control system (P-PTD 200). Prior to measurement, samples were loaded onto the plate and allowed to equilibrate for 5 min at 25 °C. Steady-state shear viscosity was measured over a shear rate range of 0.1–100 s^−1^. Dynamic oscillatory tests were subsequently performed within the linear viscoelastic region (strain amplitude = 0.1%) at 25 °C, with frequency sweep conducted from 0.1 to 10 Hz (angular frequency: 0.63–62.8 rad/s) and the gap set at 1 mm. Storage modulus (G′), loss modulus (G″), and loss tangent (tan δ = G″/G′) were recorded and analyzed.

#### 4.8.6. Cryogenic Scanning Electron Microscopy

Cryogenic laser scanning confocal microscopy (CLSM) was performed following a modified protocol adapted from Abou-Elsoud et al. [15]. Briefly, 1 mL of freshly prepared PKCG-1-based emulsion was incubated with 20 μL of Nile Blue A sulfate (1 mg/L) and 20 μL of 9-(diethylamino)-benzo-α-phenoxazin-5(5H)-one (1 mg/L, dissolved in isopropanol) in the dark for 30 min. Subsequently, 12 μL of the stained sample was deposited onto a clean glass slide, air-dried under ambient conditions, and immediately vitrified by plunge-freezing in liquid nitrogen-cooled ethane. Imaging was conducted using a Zeiss LSM 880 Airyscan system (Carl Zeiss AG, Oberkochen, Germany) equipped with cryogenic stage control. Excitation was achieved at 633 nm (for Nile Blue A) and 488 nm (for the phenoxazine derivative), respectively; emission signals were collected in sequential mode to avoid spectral crosstalk. All images were acquired at a magnification of 40×.

### 4.9. Statistical Analysis

Experiments were performed in at least triplicate. Statistical significance was assessed using a one-way analysis of variance (ANOVA) followed by Tukey’s post hoc test (IBM SPSS Statistics v14, IBM Corp., Armonk, NY, USA). The statistically significant level was set at *p* < 0.05.

## Figures and Tables

**Table 1:** Effects of ultrasonication-assisted *L*-arabinose glycosylation or gallic acid conjugation on the amino acid composition of palm kernel cake glutelin-1 (PKCG-1) (g/100 g).

Proteins	PKCG-1	PKCG-1-UGA	PKCG-1-UA
Isoleucine	3.72 ± 0.23 a	3.02 ± 0.30 a	4.03 ± 1.70 a
Leucine	7.51 ± 0.17 a	6.29 ± 0.16 b	7.83 ± 0.18 a
Lysine	6.52 ± 0.16 a	4.96 ± 0.65 a	3.52 ± 0.30 b
Methionine	2.14 ± 0.31 a	1.26 ± 0.06 b	1.97 ± 0.45 a
Phenylalanine	4.65 ± 0.29 a	3.85 ± 0.21 b	4.80 ± 0.51 a
Threonine	3.23 ± 0.04 a	3.00 ± 0.11 a	2.37 ± 0.36 b
Tryptophan	ND	ND	ND
Valine	4.80 ± 1.02 a	3.95 ± 0.08 b	4.35 ± 0.57 a
Histidine	2.54 ± 0.80 a	1.89 ± 0.11 a	2.74 ± 0.45 a
Tyrosine	5.68 ± 0.46 a	3.04 ± 0.31 c	4.59 ± 0.27 b
Aspartic acid	14.37 ± 0.29 a	14.22 ± 0.34 a	11.37 ± 0.29 a
Asparagine	5.7 5± 0.59 a	5.31 ± 0.50 a	2.89 ± 0.11 b
Serine	3.89 ± 0.34 a	3.84 ± 0.06 a	3.54 ± 0.08 a
Glutamic acid	25.01 ± 3.61 a	26.21 ± 0.88 b	21.50 ± 3.47 b
Glycine	4.54 ± 0.07 a	4.75 ± 0.31 a	4.80 ± 0.14 a
Alanine	3.93 ± 0.25 a	3.74 ± 0.35 a	4.06 ± 0.30 a
Arginine	4.25 ± 1.37 a	4.11 ± 0.27 a	3.92 ± 0.27 a
Proline	3.22 ± 0.39 a	2.45 ± 0.12 b	3.60 ± 0.50 a

Different lowercase letters (a–c) in the same row indicate significant differences (*p* < 0.05); ND, Not detected.

## Data Availability

The original contributions presented in the study are included in the article, further inquiries can be directed to the corresponding authors.

## Abbreviations

The following abbreviations are used in this manuscript:

PKCG-1	Palm kernel cake glutelin-1
PKCG-1-UA	Palm kernel cake glutelin-1 modified via ultrasonication-assisted arabinose-glycosylation
PKCG-1-UGA	Palm kernel cake glutelin-1 modified via ultrasonication-assisted gallic acid-grafting
